# Impaired neural networks for approximate calculation in dyscalculic children: a functional MRI study

**DOI:** 10.1186/1744-9081-2-31

**Published:** 2006-09-05

**Authors:** Karin Kucian, Thomas Loenneker, Thomas Dietrich, Mengia Dosch, Ernst Martin, Michael von Aster

**Affiliations:** 1 MR-Center, University Children's Hospital, Zurich, Switzerland; 2 Center for Integrative Human Physiology, University of Zurich, Switzerland; 3 Department of Child and Adolescent Psychiatry, University of Zurich, Switzerland; 4 Department of Child and Adolescent Psychiatry, DRK-Hospital Westend Berlin, Germany

## Abstract

**Background:**

Developmental dyscalculia (DD) is a specific learning disability affecting the acquisition of mathematical skills in children with otherwise normal general intelligence. The goal of the present study was to examine cerebral mechanisms underlying DD.

**Methods:**

Eighteen children with DD aged 11.2 ± 1.3 years and twenty age-matched typically achieving schoolchildren were investigated using functional magnetic resonance imaging (fMRI) during trials testing approximate and exact mathematical calculation, as well as magnitude comparison.

**Results:**

Children with DD showed greater inter-individual variability and had weaker activation in almost the entire neuronal network for approximate calculation including the intraparietal sulcus, and the middle and inferior frontal gyrus of both hemispheres. In particular, the left intraparietal sulcus, the left inferior frontal gyrus and the right middle frontal gyrus seem to play crucial roles in correct approximate calculation, since brain activation correlated with accuracy rate in these regions. In contrast, no differences between groups could be found for exact calculation and magnitude comparison. In general, fMRI revealed similar parietal and prefrontal activation patterns in DD children compared to controls for all conditions.

**Conclusion:**

In conclusion, there is evidence for a deficient recruitment of neural resources in children with DD when processing analog magnitudes of numbers.

## Background

The present study was aimed at investigating the neural underpinnings of developmental dyscalculia in schoolchildren using functional magnetic resonance imaging (fMRI).

Specific disorders of numerical skills are neither widely recognized nor well understood. Children can exhibit low math performance in many different ways [1]. Some may have particular difficulties with arithmetical facts [2], others with procedures and strategies [3], while most disabled children seem to have difficulties across the whole spectrum of numerical tasks [4]. Just as diverse as the manifestation of specific math learning disabilities is the wide range of terms referring to these developmental math disabilities (developmental dyscalculia, mathematical disability, arithmetical learning disability, number fact disorder, psychological difficulties in mathematics). The term 'developmental dyscalculia' (DD) will be used here, which is defined as a significant discrepancy between specific math performance and performance in other domains (i.e. reading) and/or general intelligence that cannot be explained by mental retardation, inappropriate schooling or poor social environment according to the International Classification of Diseases, 10^th ^revision (ICD-10, F81.2, [5]). Prevalence studies using different definitions of DD have been carried out in various countries. In spite of the lack of definitional consistency, the prevalence of DD across countries is relatively uniform, ranging from 3–6% in the normal population, which is similar to that of developmental dyslexia and attention deficit hyperactivity disorder (ADHD) [6]. Unlike these other learning disabilities, girls and boys seem to be affected by DD equally [6,7]. Furthermore, DD seems to be an enduring specific learning difficulty, persisting into late adolescence [8]. While it is clearly the case that DD is frequently co-morbid with a variety of disorders, like dyslexia, ADHD, poor hand-eye coordination, poor working memory span, epilepsy, fragile X syndrome, Williams syndrome and Turner syndrome, causal relationships between these disorders have not been established [1,9-13]. For illustration, about one quarter of dyscalculics show comorbidity with ADHD and dyslexia [10,13]. Over the past two decades sufficient genetic, neurobiological, and epidemiologic evidence has accumulated to indicate that learning disabilities, including DD, are in fact expressions of brain dysfunction [12-18].

Functional neuroimaging has revealed that parietal and prefrontal cortices are involved in arithmetic tasks [19-24]. In particular, the intraparietal sulcus (IPS) seems to play a major role in number processing [25]. While in adults the IPS seems to be mainly involved in the processing of numerical quantities, number comparison and simple quantity manipulations, including approximation, the left angular gyrus and left prefrontal regions are mainly implicated in exact, verbal memory based, language-dependent calculation [20,26,27]. According to this functional dissociation, circumscribed lesions should cause different patterns of dysfunction depending on whether the lesion affects the quantity or the verbal system of numerical representation [28]. Indeed, patients with IPS lesions particularly fail to comprehend numerical quantities that are required in approximation, numerical comparison tasks or solving subtraction trials, whereas simple multiplication might be unaffected, presumably because it can still be retrieved from intact verbal memory [29-31]. Conversely, patients with acalculia following a perisylvian lesion exhibited greater impairment in the verbal processing of numbers needed for multiplication tables rather than for quantity-based operations [29,32]. Although several imaging as well as lesion studies corroborate the existence of a dissociated processing for exact and approximate calculation [24,29,31], this dissociation could not be systematically replicated in normal and disabled calculators [33,34]. Moreover, studies testing fact retrieval based on multiplication or addition and quantity processing by using subtraction or number comparison tasks could also not identify a neuronal dissociation. In contrast, they found close activation patterns for verbal and quantity based processing of numbers [22,35].

Do children and adults with DD exhibit the same neuronal networks reported in typically achieving subjects? Can dissociation between the verbal and the quantity-based system be observed in children and adults with DD? Is one of these systems more affected by DD? These are only a few of the open questions in dyscalculia research. Answers to these questions would enable us to specify the neuronal underpinnings of this learning disability and provide new possibilities to develop and evaluate therapeutic interventions.

Thus far, a few MRI studies have begun to elucidate the correlation of dyscalculia with neuronal processing, morphology and metabolism in patients with dyscalculia. Functional [34,36-38], spectroscopic [38] and morphometric [39,40] MRI techniques have been conducted on dyscalculia.

FMRI has been used to study neural activity in a subject with dyscalculia secondary to a right temporal lobe hemorrhage endured during infancy [37,38], and in samples of patients with Turner's [34] and fragile X syndrome [36]. All these fMRI studies provide evidence for an abnormality in the parietal cortices with overall diminished as well as abnormally modulated IPS activity, correlating to number size or task difficulty [34,36,37]. Investigating verbal and quantity based number processing, Molko et al. [34] used an experimental design that included exact and approximate addition tasks in subjects with Turner's syndrome. In contrast to controls, these subjects failed to show any difference in brain activation between trials of exact and approximate calculation, although they performed the calculations as well as the controls. Molko and colleagues concluded that Turner's syndrome subjects might have used quantity manipulation strategies during both tasks [34].

By means of magnetic resonance spectroscopy a defect in the left parieto-temporal area was indicated in one patient with DD without any known structural abnormality [38].

Voxel-based morphometry indicated a decrease in grey matter density in the left IPS in premature children with DD [40] and in a symmetrical location in the right IPS of Turner syndrome subjects [39]. In addition, morphometric analysis revealed atypical anatomy of the right IPS in Turner syndrome subjects [39].

In summary, the data derived from findings in patients with dyscalculia suggest that defects in parietal areas seem to be particularly responsible for arithmetic problems. However, the potentially distinct roles of the left and right IPS remain to be clarified. Finally, the question remains open whether observed structural abnormalities in IPS are the cause or consequence of poor arithmetic ability [1].

The present fMRI study investigated brain activation in 18 children with DD and 20 typically achieving schoolchildren during different numerical tasks. We used exact and approximate addition tasks that examine the verbal and the quantity based system in cerebral representations of numbers. The comparison of exact and approximate calculation is expected to assess a dissociation of these processes and afford a more specialized delineation of possible functional deficits in children with DD. Additionally, non-symbolic magnitude comparison was used to investigate basic number representations independent of the Arabic notation system. The present study represents the first examination of brain activation in a non-clinical sample of children with DD. Therefore, it is difficult to pose clear hypotheses. However, according to previous findings and the important role of the parietal lobe in number processing, we expect that children with DD would show deviations in parietal activation patterns compared to typically achieving children. In particular, differences in IPS activation during approximate calculation are anticipated since the IPS is supposed to represent the neuronal correlate of the mental number line in typical calculators. Moreover, an existing fMRI study in dyscalculic patients provided evidence for an abnormal activation of this region during approximate calculation [34]. With respect to neuropsychological theory of verbal and quantity based representations [27], we predict that children with DD might show a disorganization of the functional patterns and stronger impairments of quantity based representations compared to verbal representations. However, whether such a dissociation could really be expected is still unclear, as the literature reports divergent findings.

## Methods

### Subjects

Eighteen children with DD and 20 typically achieving schoolchildren participated in this fMRI-study. Participants were healthy, right-handed volunteers with no psychiatric or medical complications as determined by a detailed questionnaire. None of the children suffered from any neuroanatomical abnormalities as determined by high-resolution structural magnetic resonance scans. All participants were medication-free.

Typically achieving schoolchildren in two age ranges were examined: younger subjects attending the 3^rd ^grade (5 females and 5 males, mean age 9.2 ± 0.2 years) and older children attending the 6^th ^grade (5 females and 5 males, mean age 12.0 ± 0.3 years).

Eighteen age-matched children with DD were carefully selected (younger group: 5 females and 4 males, mean age 10.1 ± 0.6 years; older group: 9 females, mean age 12.3 ± 0.6 years). An independent samples t-test confirmed that dyscalculic and typically achieving children were in the same age range (p < 0.3). It was not possible to balance gender in the DD group because fewer boys volunteered and most of them did not fulfill participation criteria. The high number of volunteering girls likely reflects the relative predominance of girls with DD [6,7]. In addition, our previous study showed no gender differences in typically achieving children performing the same number processing tasks [41].

Written, informed consent for the participation in this study was obtained from the legal guardians of the children. The study was approved by the local ethics committee based on the World Medical Association's Declaration of Helsinki [42].

### Behavioral tests in children

Behavioral tests in children with DD were executed by a trained specialist, e.g. from the psychological school services. Batteries included tests to assess their mathematical, linguistic and spatial abilities as well as their IQ (examples: ZAREKI, [43]; K-ABC, [44]; HAWIK-III, [45]). Dyscalculia was clearly diagnosed in all of our subjects. The diagnosis was based on the definition of the ICD-10 [5], which uses the discrepancy between the individual's general intelligence and his or her mathematical performance that cannot be explained by inadequate schooling, sensory deficits or other neurological, psychiatric or medical disorders alone. All children had an IQ above 80. None of these children suffered from any other neurological, psychiatric or learning disorders (e.g. dyslexia, ADHD).

All typically achieving children were made to undergo behavioral tests to assess their linguistic and mathematical competence prior to the study. Children in the 6^th ^grade completed two modules of a test battery for semantic and linguistic verbal fluency in German and mathematical competence [46]. All 6^th ^grade children showed average age-related performance in both modules compared to the 6^th ^grade Swiss normative sample of over 500 children, indicated in italics (verbal score: 37.1 (5.5); *N = 512, 38.1 (6.84)*; mathematical score: 16.1 (4.0); *N = 517, 14.2 (4.14)*). Children in the 3^rd ^grade were tested for number processing and calculation abilities (ZAREKI, [43]) and for reading and spelling skills (Knuspel's Leseaufgaben, [47]; Salzburger Lese- und Rechtschreibtest, [48]). All children showed normal age-related performance compared to a Swiss normative sample of 337 age-matched children, indicated in italics (ZAREKI: 147.5 (21.9) ; *143.6 (27.7)*; Knuspel's Leseaufgaben: 26.5 (2.9); *21.2 (8.6)*; Salzburger Lese- und Rechtschreibtest: 8.0 (1.7); *7.53 (4.2))*.

### Stimuli and task

Before entering the scanner the participants were carefully instructed about the examination procedure and task. First, the paradigm was explained and performed on a desktop computer outside the scanner and children had to solve one to three 20 s blocks of practice trials by pressing the corresponding mouse button. After completing practice trials with a minimum of 60% correct answers within a block, we proceeded to the actual fMRI experiment.

**Figure 1:**
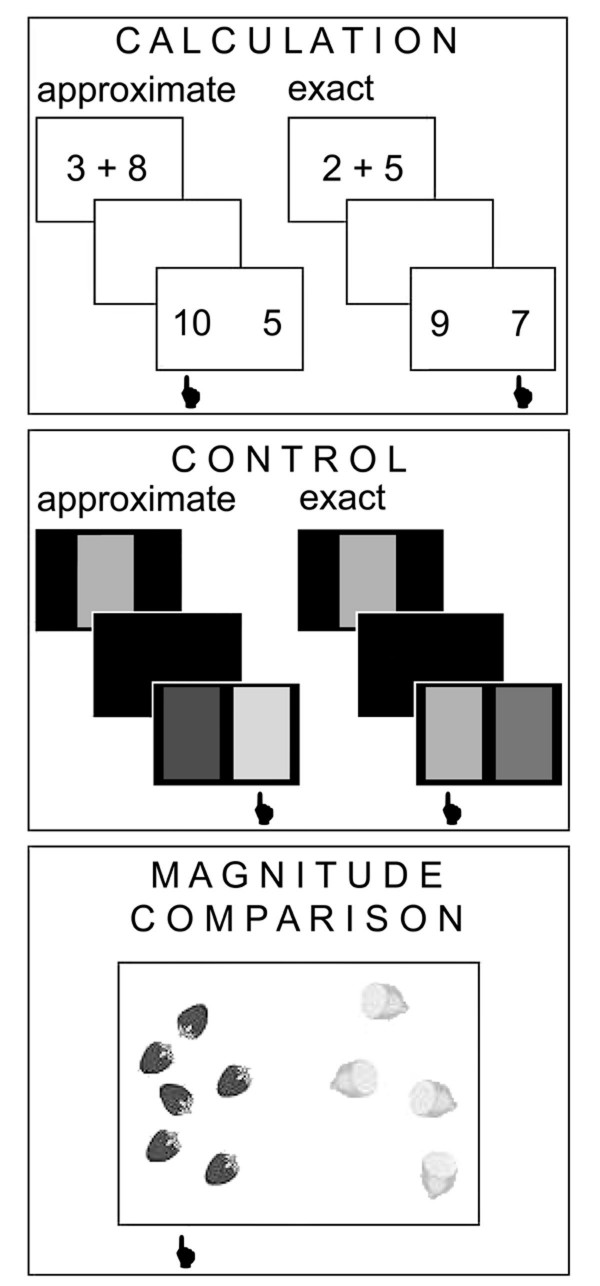
**Paradigm**. The paradigm used during fMRI examination consisted of approximate and exact calculation, approximate and exact control conditions as well as magnitude comparison. Each condition was presented in three blocks of 80 s.

During fMRI examination the computer-generated paradigm (E-Prime, Psychology Software Tools Inc.) was presented to the subjects via video-goggles (Resonance Technology Inc., Northridge, U.S.A.) and synchronized with the fMRI sequence using the scanner trigger pulses. Behavioral data were collected by means of a response box (LUMINA, Cedrus Corporation, San Pedro, U.S.A). The paradigm was a classical boxcar design consisting of three experimental conditions: (1) approximate and exact calculation, (2) approximate and exact control condition, (3) magnitude comparison of small numbers of objects (Fig. 1).

Each block lasted 80 s separated by a break of 12.5 s. During the rest condition, subjects were asked to focus on a fixation mark at the center of the screen. The three conditions and trials within a block were presented to all subjects in a randomized order. The number of trials within one block varied between subjects due to the self-paced stimulation. Self-paced stimulation allows solving the tasks on each individual working load and thus provokes in typically achieving and dyscalculic children strong brain activation of corresponding regions.

#### Calculation

The calculation task consisted of three cycles of alternating approximate (AP) and exact (EX) calculation blocks. First, a single-digit addition was presented for 850 ms, followed by a break of 200 ms and two alternative solutions again presented during 850 ms. Afterwards, subjects had a maximum time of 6 s to give their answer. The subsequent trial started 1 s after the previous response. In the exact addition condition, subjects selected the correct sum of two numerically close numbers by pressing a response button. In the approximate addition condition, they were asked to estimate the result and to select the closest number of two choices.

#### Control condition

The control condition for the calculation trials was a grayscale discrimination task, again presented during three cycles of approximate and exact discrimination blocks using an equal stimulus presentation time and inter-stimulus interval (ISI). Stimuli were presented in randomized order. In the exact control task, subjects had to match sequentially presented grayscale patterns. In the approximate control task, they were asked to pick the grayscale pattern with the most similar luminosity. Alternative solutions were more alike in the exact control condition than those in the approximate control condition.

#### Magnitude comparison

The magnitude comparison (MC) task involved three blocks of 80 s. Subjects had to compare two sets of different objects (pictures of fruit or vegetables) and were asked to select the set with the larger number of objects. The maximal duration of stimulus presentation was 2 s and the ISI was 1 s. The maximum number of objects displayed on one side was 18. The differences between the two sets were 1, 2, 3 or 4 in the first block; 9, 10, 11 or 12 in the second block; and 5, 6, 7 or 8 in the third block. Fixation during rest served as the control condition for magnitude comparison.

### Magnetic resonance imaging

Functional MRI acquisition was performed on a 1.5 Tesla whole-body system (GE Medical Systems, Milwaukee, WI, USA), using gradient-recalled echo planar imaging (repetition time, TR = 3.2 s; echo time, TE = 55 ms; field of view (FOV) = 240 mm × 240 mm; flip angle = 90°C; matrix size = 64 × 64; voxel size = 3.75 mm × 3.75 mm; 32 contiguous slices parallel to the AC-PC line, slice thickness = 4 mm). Three-dimensional anatomical images of the entire brain were obtained by using a T1-weighted gradient echo pulse sequence (TR = 27 ms; FOV = 240 mm × 240 mm × 144 mm; matrix size = 256 × 192 × 90).

### Data analysis

Statistical analysis of behavioral data was based on 18 children with DD and 20 typically achieving children using SPSS [49].

FMRI-data were analyzed using the Statistical Parametric Mapping (SPM99) software (Wellcome Department of Cognitive Neurology, London, UK). The first four images were discarded thus allowing for a steady-state magnetization. All images were realigned and transformed into the standardized stereotactic reference system (EPI-template provided by the Montreal Neurological Institute). Absolute motion was less than half a voxel size for all subjects. Each normalized scan was smoothed with a 9 mm full-width at half-maximum Gaussian kernel. Changes in regional blood oxygen level-dependent (BOLD) contrast were determined by applying the general linear model (GLM) to each voxel. A within group voxel-wise comparison of BOLD response was performed using t-statistics to test for significant changes in BOLD contrast. The resulting set of voxel values for each contrast constituted a statistical parametric map of the t-statistic (SPM(T)). A second level analysis was performed on the basis of the linear contrasts for each subject and condition. Reported activated brain regions of random effects analysis had been subjected to a family-wise error (FWE) correction with a minimum number of 10 voxels. If no activation cluster bore up under FWE-correction the more liberal false discovery rate (FDR) correction was applied [50]. For two-sample t-test analysis uncorrected thresholds were also consulted. Finally, MNI coordinates (Montreal Neurological Institute) of activated voxels were transformed into the Talairach and Tournoux reference system using the MNI2TAL tool (MNI2TAL, Matthew Brett). Localization of Talairach coordinates was performed by Talairach Daemon [51] and Talairach atlas [52].

Region of interest (ROI) definition, extraction of data for the region and statistical analysis of ROI data using the SPM statistics machinery were performed by MarsBar (MarsBar Version 0.37, Matthew Brett). ROIs were defined by significantly activated clusters in the random effect analysis of typically achieving children for all conditions at a FWE corrected level of *p *< 0.05. Only ROIs containing more than 10 voxels were included. Table 1 summarizes all defined ROIs, the corresponding range of Talairach coordinates and the volume of ROIs for all conditions. The percentage of signal change (ΔS) and mean t-values were computed within these ROIs for all subjects.

**Table 1:** Defined regions of interest (ROIs)

**Condition**	**Talairach coordinates**			***Volume (mm***^***3***^***)***
	***x***	***y***	***z***	

Approximate calculation				
Left intraparietal sulcus (IPS)	-45 to -6	-80 to -35	32 to 52	6588
Right intraparietal sulcus (IPS)	18 to 36	-78 to -53	23 to 50	4401
Left inferior frontal gyrus (IFG)	-39 to -27	14 to 26	-8 to 4	1458
Right inferior frontal gyrus (IFG)	24 to 45	14 to 33	-13 to 9	4023
Left middle frontal gyrus (MFG)	-33 to -18	-10 to 9	42 to 60	2916
Right middle frontal gyrus (MFG)	27 to 50	27 to 51	12 to 31	4536
Anterior cingulate gyrus (ACG)	-12 to 15	2 to 29	30 to 54	5535

Exact calculation				
Left inferior frontal gyrus (IFG)	-39 to -27	15 to 30	-1 to 12	1161
Left fusiform gyrus (FG)	-48 to -36	-75 to -55	-16 to -2	1448
Left intraparietal sulcus (IPS)	-42 to -24	-65 to -44	36 to 52	2295
Right intraparietal sulcus (IPS)	24 to 51	-74 to -38	26 to 46	3504

Magnitude comparison				
Left fusiform gyrus (FG)	-45 to -6	-97 to -43	-23 to 5	6480
Right fusiform gyrus (FG) & right middle occipital gyrus (MOG)	6 to 44	-94 to -48	-21 to 21	6376
Right intraparietal sulcus (IPS)	21 to 30	-77 to -62	37 to 47	1323

Defined ROIs for each condition, corresponding range of Talairach coordinates and volume of ROI are listed.

## Results

### Behavioral results

Table 2 shows the mean number of trials, reaction times, accuracy rates and the corresponding standard deviations for the different conditions in children with and without DD. Completed number of trials did not differ between children with or without dyscalculia in any condition (AP: *p *> 0.5; EX: *p *> 0.7; MC: *p *> 0.2). For each condition, a multivariate GLM was computed with the reaction time (RT) and accuracy rate (ACC) as dependent variables and type (with or without DD), grade (3^rd ^or 6^th ^grade) or gender (female or male) as a fixed factor.

**Table 2:** Behavioral results

Conditions	Number of trials, Mean (S.D.)		3^rd ^grade children		6^th ^grade children	
	
	Control children	Dyscalculic children	Control children	Dyscalculic children	Control children	Dyscalculic children
***Approximate Calculation***	72.8 (13.2)	70.2 (12.2)				
Reaction time (ms)						
Mean (S.D.)			1659 (660)	1467 (523)	891 (352)	929 (351)
Accuracy rate (%)						
Mean (S.D.)			79.5 (4.9)	68 (17.0)	89.5 (4.6)	86 (9.2)
***Exact calculation***	73.6 (16.1)	72.2 (14.0)				
Reaction time (ms)						
Mean (S.D.)			1707 (763)	1499 (494)	768 (370)	872 (426)
Accuracy rate (%)						
Mean (S.D.)			73.7 (8.2)	60 (17.2)	87.9 (8.3)	84 (10.2)
***Magnitude comparison***	127.3 (15.5)	121.5 (14.7)				
Reaction time (ms)						
Mean (S.D.)			931 (254)	964 (288)	702 (176)	827 (189)
Accuracy rate (%)						
Mean (S.D.)			97.2 (2.0)	95 (1.9)	96.7 (1.3)	97 (1.6)

Means and standard deviations (SD) of the number of trials, reaction times and accuracy rates for 3^rd ^grade and 6^th ^grade children with or without DD during approximate or exact calculation and magnitude comparison are presented.

Wilks' lambda tests revealed significant differences between 3^rd ^and 6^th ^graders (F (6, 31) = 7.519, *p *< 0.001). Post-hoc t-tests indicated significantly higher ACC rates for 6^th ^graders in approximate (*p *< 0.001) as well as exact calculation (*p *< 0.001), and a trend in the same direction was observed in magnitude comparison (*p *< 0.1). In addition, children in the 3^rd ^grade answered significantly slower than 6^th ^grade children under all conditions (AP: *p *< 0.001; EX: *p *< 0.001; MC: *p *< 0.05). The improvement in performance with age is also reflected in significant Pearson correlations of ACC and RT with age (AP: ACC r = 0.387, *p *< 0.05; RT r = -0.694, *p *< 0.01/EX: ACC r = 0.497, *p *< 0.01; RT r = -0.710, *p *< 0.01/MC: RT r = -0.433, *p *< 0.01).

Multivariate GLM analysis revealed no significant differences either between children with or without DD (F (6, 31) = 1.188, *p *< 0.4) or between the genders (F (6, 31) = 0.958, *p *< 0.5). Additionally, calculated effect sizes (Cohen's d, [53]) for differences between children with or without DD as well as for gender differences ranged from small values to medium values for both effects (with or without DD: min. d = .08 (EX RT), max. d = .64 (AP ACC); gender differences: min. d = 0.1 (ACC AP), max. d = 0.57 (RT MC). Furthermore, statistical power analysis was performed for MANOVA using SPSS including values for mean, correlations and standard deviations calculated from the present data [54]. Statistical power for differences between children with or without DD (ACC: power = .30; RT: power = .19) and between the genders was small (ACC: power = .08; RT: power = .25). Due to the small effect sizes and power of statistical differences between groups and genders, post-hoc t-tests were conducted. Like the results of the multivariate GLM analysis, none of these t-tests between children with or without DD, or between genders, reached significance for any condition (p > 0.05).

To test for differences between conditions, a repeated-measures GLM analysis was calculated with RT or ACC for exact calculation, approximate calculation and magnitude comparison as within-subjects factors, and type (with or without DD) as between-subjects factor. Statistical Wilks' lambda analysis indicated significant differences between conditions for RT (F (2, 35) = 10.932, *p *< 0.01) as well as for ACC (F (2, 35) = 39.616, *p *< 0.01). Interactions between conditions and type turned out to be not significant for RT and ACC. Post-hoc t-tests between conditions demonstrated that both typically achieving and DD children performed most accurately and rapidly in the magnitude comparison condition compared to calculation conditions (ACC: typical children: *p *< 0.001; children with DD: *p *< 0.001; RT: typical children: *p *< 0.001; children with DD: *p *< 0.01). Neither for children with nor for those without DD, did RT and ACC differ between approximate and exact calculation.

### Functional MRI results

In general, similar cerebral patterns were activated in children with and without DD, including parietal and prefrontal regions. However, the observed brain activation patterns in children with DD were more diffuse and showed greater inter-individual variance. Hence, mean t-values of activated voxels were lower in children with DD compared to typically achieving children.

#### Random-effects analysis

**Figure 2:**
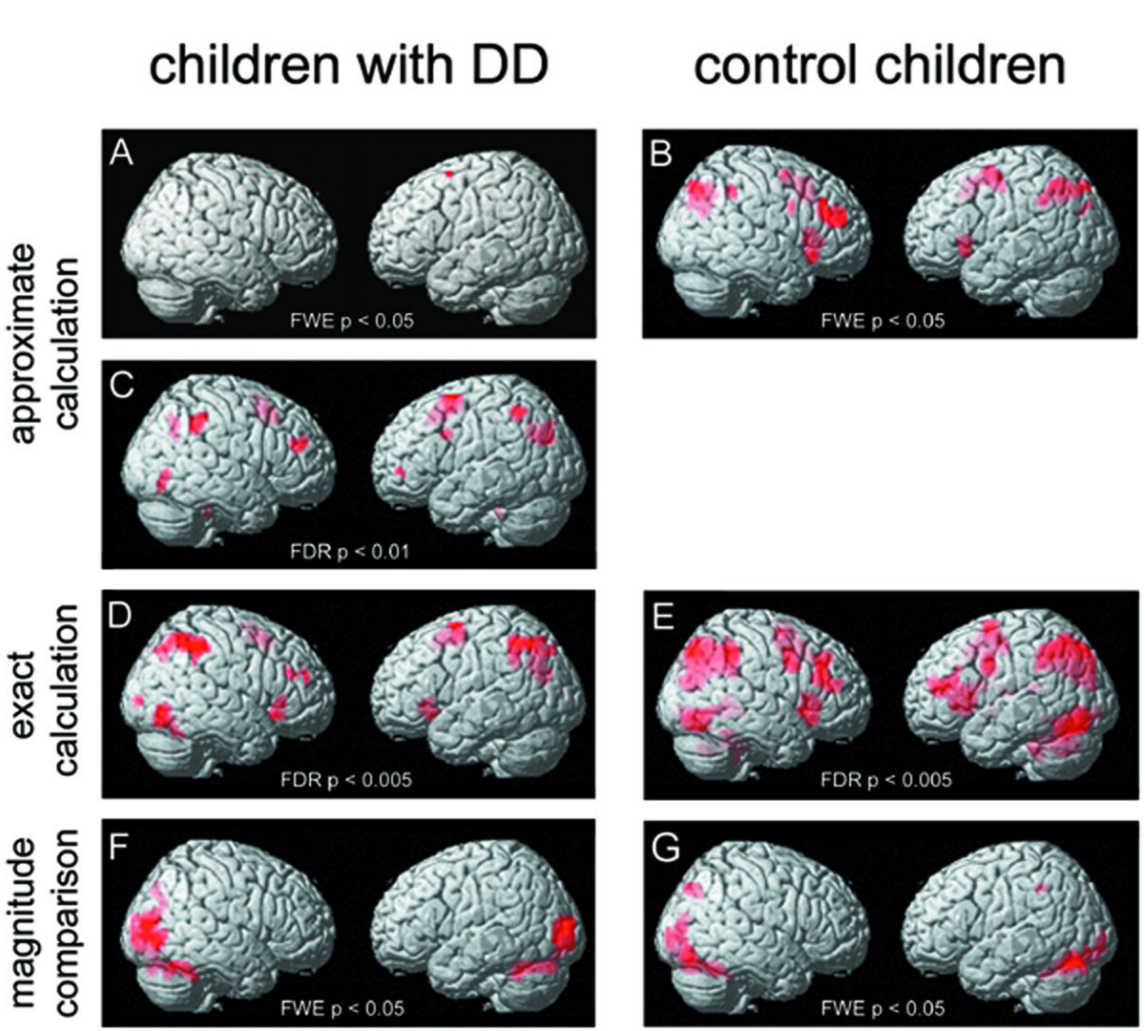
**Brain activation of children with DD and control children**. Brain activation patterns of children with DD (N = 18) and control children (N = 20) during each condition are depicted on the SPM standard brain template. The activated brain regions shown had been subjected to a FWE or FDR correction with a minimum number of 10 voxels, with one exception in Figure 2C, where the shown cluster comprises only 5 voxels. A, B, C: approximate calculation – approximate control condition. D, E: exact calculation – exact control condition. F, G: magnitude comparison – rest.

Figure 2 shows activation patterns in children with and without DD during all three conditions. Since contrasts between 3^rd ^and 6^th ^grade children showed no significant differences in brain activation in any condition, the reported group analysis includes all 18 dyscalculic and all 20 control subjects.

#### Approximate calculation versus approximate control condition (Table 3)

**Table 3:** Cortical activation in children with DD and control children during approximate calculation

**Anatomical region**	**Talairach coordinates**			***T***	**Number of voxels in cluster**
	***x***	***y***	***z***		
*Children with DD (p < 0.01 FDR corrected)*					
Left middle frontal gyrus (MFG)	-27	6	58	7.48	92
Left intraparietal sulcus (IPS)	-27	-71	31	6.69	108
Bilateral anterior cingulate gyrus (ACG)	-3	14	44	6.56	162
Left middle frontal gyrus (MFG)	-39	47	-2	6.33	13
Left inferior parietal lobe (IPL)	-45	-47	49	6.31	36
Right intraparietal sulcus (IPS)	45	-39	38	6.23	77
Right middle frontal gyrus (MFG)	42	39	17	5.91	28
Right middle occipital gyrus (MOG)	45	-75	-12	5.41	27
Left inferior frontal gyrus (IFG)	-45	10	30	5.59	15
Right intraparietal sulcus (IPS)	33	-59	42	5.27	28

*Control children (p < 0.05 FWE corrected)*					
Left middle frontal gyrus (MFG)	-27	0	53	15.03	108
Bilateral anterior cingulate gyrus (ACG)	12	16	38	11.8	205
Left intraparietal sulcus (IPS)	-30	-53	44	9.59	244
Left inferior frontal gyrus (IFG)	-36	20	-6	9.7	54
Right inferior frontal gyrus (IFG)	39	23	-9	9.41	149
Right intraparietal sulcus (IPS)	33	-65	45	9.41	163
Right middle frontal gyrus (MFG)	33	39	20	9.1	168
Left precuneus	-24	-77	34	8.34	15
Right inferior parietal lobe (IPL)	48	-39	41	8.16	24
Right middle frontal gyrus (MFG)	30	0	53	8.12	23
Right inferior frontal gyrus (IFG)	48	7	30	7.83	14

Anatomical localization, Talairach coordinates, *T *scores of significant activated voxels and cluster size of activated regions with a minimum number of 10 voxels in children with DD (FDR *p *< 0.01) and control children (FWE *p *< 0.05) during approximate calculation versus approximate control condition are listed.

Children with developmental dyscalculia

Children with DD showed almost no activation when using FWE correction for approximate calculation vs. approximate control condition (see Figure 2A). By means of a less strict correction (FDR), they exhibited significant (*p *< 0.01 *FDR corrected*) bilateral activation during approximate calculation in the middle frontal gyrus (MFG), intraparietal sulcus (IPS) and anterior cingulate gyrus (ACG). Unilateral activation was found in the left inferior parietal lobe (IPL), right middle occipital gyrus (MOG) and left inferior frontal gyrus (IFG) (see Figure 2C).

Control children

The brain activation pattern of typically achieving children (*p *< 0.05 *FWE corrected*) during approximate calculation resembled those of the children with DD. Yet activated clusters reached higher mean t-values compared to mean t-values of activation in children with DD (*p *< 0.001). The activated network included the middle frontal gyrus (MFG), the anterior cingulate gyrus (ACG), the intraparietal sulcus (IPS) and the inferior frontal gyrus (IFG) in both hemispheres. Additionally, activation was found in the left precuneus and right inferior parietal lobe (IPL) (see Figure 2B).

#### Exact calculation versus exact control condition (Table 4)

**Table 4:** Cortical activation in children with DD and control children during exact calculation

**Anatomical region**	**Talairach coordinates**			***T***	**Number of voxels in cluster**
	***x***	***y***	***z***		
*Children with DD (p < 0.005 FDR corrected)*					
Right middle occipital gyrus (MOG)	45	-73	-6	7.36	94
Right inferior temporal gyrus (ITG)	45	-70	1	6.0	
Right fusiform gyrus (FG)	45	-59	-17	5.7	
Left intraparietal sulcus (IPS)	-45	-47	50	6.57	288
Right inferior frontal gyrus (IFG)	21	-90	7	6.95	20
Right intraparietal sulcus (IPS)	48	-36	46	6.82	242
Right inferior parietal lobe (IPL)	53	-41	49	6.72	
Anterior cingulated gyrus (ACG)	0	8	49	6.3	120
Left inferior frontal gyrus (IFG)	-30	23	-1	6.22	54
Right inferior frontal gyrus (IFG)	36	23	-11	6.08	76
Left middle frontal gyrus (MFG)	-27	3	58	5.8	98
Right middle frontal gyrus (MFG)	39	36	20	5.59	15
Right middle frontal gyrus (MFG)	42	48	20	5.36	12

*Control children (p < 0.005 FDR corrected)*					
Left inferior frontal gyrus (IFG)	-33	26	1	7.42	491
Left intraparietal sulcus (IPS)	-30	-56	44	10.01	902
Right superior parietal lobe (SPL)	30	-71	45	9.33	914
Right intrapaprietal sulcus (IPS)	45	-42	41	8.63	
Left precentral gyrus	-48	2	33	8.8	126
Right inferior frontal gyrus (IFG)	36	18	7	8.49	211
Left middle occipital gyrus (MOG)	-45	-73	-4	7.78	806
Left fusiform gyrus (FG)	-39	-59	-12	7.54	
Left superior frontal gyrus (SFG)	-24	3	61	6.99	481
Left middle frontal gyrus (MFG)	-27	-9	47	6.41	
Right inferior occipital gyrus (IOG)	42	-79	-6	6.46	238
Right inferior temporal gyrus (ITG)	48	-67	-2	5.67	
Right inferior frontal gyrus (IFG)	50	7	33	6.27	93
Right middle frontal gyrus (MFG)	42	37	37	4.51	214
Right middle frontal gyrus (MFG)	27	0	58	5.35	68
Left thalamus	-12	-14	12	5.17	36
Right lingual gyrus (LG)	18	-79	-1	5.16	27
Left lingual gyrus (LG)	-18	-87	-1	4.72	17
Right fusiform gyrus (FG)	45	-47	-15	4.66	13

Anatomical localization, Talairach coordinates, *T *scores of significant activated voxels and cluster size of activated regions in children with DD and control children during exact calculation versus exact control condition are specified. Listed activated brain regions had been subjected to a FDR correction at p < 0.005 with a minimum number of 10 voxels.

Children with developmental dyscalculia

In general, the observed activation pattern for exact calculation was quite similar to that of approximate calculation. In children with DD (*p *< 0.005 *FDR corrected*), exact calculation activated primary and secondary visual areas in the right hemisphere (MOG, inferior temporal gyrus (ITG), fusiform gyrus (FG)) and in both hemispheres the parietal lobe including the intraparietal sulcus (IPS) as well as prefrontal regions (IFG, MFG, ACG) (see Figure 2D).

Control children

In typically achieving children (*p *< 0.005 *FDR corrected*), the most intense activity was observed in the left inferior frontal gyrus (IFG). In addition, activation in the frontal lobe was found in the left precentral gyrus, left superior frontal gyrus (SFG), right inferior and middle frontal gyrus (IFG, MFG). Bilateral parietal activation was seen in the superior parietal lobe (SPL) including the intraparietal sulcus (IPS). Furthermore, primary and secondary visual areas were activated (MOG, inferior occipital gyrus (IOG), ITG, lingual gyrus (LG), FG) (see Figure 2E).

#### Magnitude comparison versus rest (Table 5)

**Table 5:** Cortical activation in children with DD and control children during magnitude comparison

**Anatomical region**	**Talairach coordinates**			***T***	**Number of voxels in clusters**
	***x***	***y***	***z***		
*Children with DD (p < 0.05 FWE corrected)*					
Right middle occipital gyrus (MOG)	33	-78	15	16.29	979
Right fusiform gyrus (FG)	30	-68	-19	12.94	
Right lingual gyrus (LG)	9	-87	-1	12.35	
Left fusiform gyrus (FG)	-27	-65	-19	10.92	
Left cuneus	-15	-87	10	10.71	
Left middle occipital gyrus (MOG)	-30	-87	4	10.57	
Right superior parietal lobe (SPL)	21	-74	40	8.35	

*Control children (p < 0.05 FWE corrected)*					
Left fusiform gyrus (FG)	-39	-68	-12	11.32	339
Left lingual gyrus (LG)	-9	-93	0	9.22	
Right cuneus	18	-93	7	10.18	418
Right fusiform gyrus (FG)	27	-71	-19	9.94	
Right intraparietal sulcus (IPS)	27	-71	42	8.76	46
Left intraparietal sulcus (IPS)	-42	-39	41	7.82	11

Anatomical localization, Talairach coordinates, *T *scores of significant activated voxels and cluster size of activated regions in children with DD and control children during magnitude comparison versus rest are listed. Activated brain regions had been subjected to a FWE correction at p < 0.05 with a minimum number of 10 voxels.

Children with developmental dyscalculia

During magnitude comparison, children with DD (*p *< 0.05 *FWE corrected*) activated a network of primary and secondary visual areas including middle occipital gyrus (MOG), fusiform gyrus (FG), lingual gyrus (LG) and cuneus. In the right hemisphere, the network extended into the parietal lobe along with the intraparietal sulcus (IPS) (see Figure 2F).

Control children

Peak locations of brain activation in control children (p < 0.05 FWE corrected) included the same regions found in children with DD. However, control children showed bilateral parietal activation foci in the intraparietal sulcus (IPS) (see Figure 2G).

#### Two-sample t-tests

##### Approximate versus exact calculation and vice versa

Contrasting approximate and exact calculation in DD children and control children by paired t-tests revealed no significant differences in activation between these two tasks when correcting the results for FWE or FDR. Using an uncorrected threshold of p < 0.0001, activation differed in clusters of more than 10 voxels between approximate and exact calculation in control children, but not in DD children.

In control children, stronger activation was found in the anterior cingulate gyrus for approximate calculation compared to exact calculation. For exact calculation stronger activation was exhibited in the left cerebellum compared to approximate calculation (see Table 6).

**Table 6:** Exact versus approximate calculation and vice versa

**Anatomical region**	**Talairach coordinates**			***T***	**k**_**E**_
	***x ***	***y***	***z***		
*Control children (p = 0.0001 uncorrected, k ≥ 10)*					
*Approximate vs. exact calculation*					
Anterior cingulate gyrus (BA32)	9	19	38	5.72	60
*Exact vs. approximate calculation*					
Left cerebellum	-15	-57	-30	6.24	13

Anatomical localization, Talairach coordinates, *T *scores and cluster size of significant differences between approximate and exact calculation in control children at *p = 0.0001 uncorrected, k ≥ 10*.

##### Dyscalculic children versus control children and vice versa

**Figure 3:**
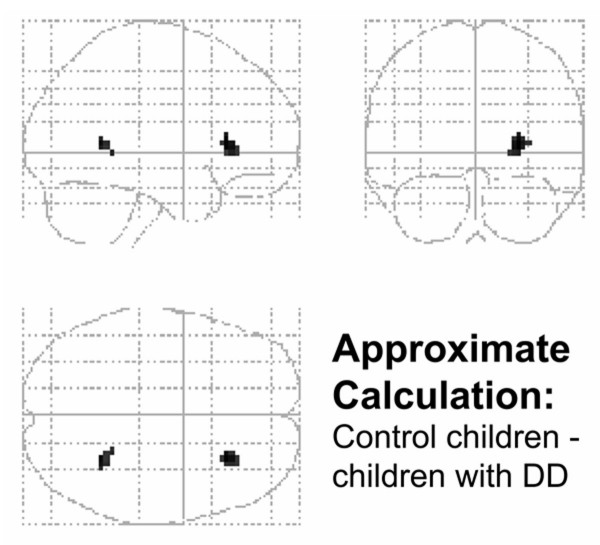
**Stronger activation in control children compared to children with DD**. Control children exhibited stronger activation in the insula and parahippocampal gyrus on the right hemisphere during approximate calculation compared to children with DD. Activated brain regions were uncorrected at p < 0.001 with a minimum number of 10 voxels represented on the SPM glass brain.

A contrast of children with DD and typically achieving schoolchildren revealed no differences either in approximate or in exact calculation or in magnitude comparison when correcting for multiple comparisons (FWE or FDR). Using uncorrected p-values of 0.001 confirm this finding, no differences could be found in brain activation between both groups during exact calculation and magnitude comparison, only during approximate calculation did control children exhibit stronger activation in two small clusters (see Table 7 and Figure 3).

**Table 7:** Direct comparison of cortical activations between children with DD and control children during approximate calculation

**Anatomical region**	**Talairach coordinates**			***T***	**Number of voxels in clusters**
	***x***	***y***	***z***		
*Control children vs. children with DD (p < 0.001 uncorrected)*					
Right Insula	27	26	1	4.20	18
Right parahippocampal gyrus	27	-49	8	3.88	13

*Children with DD vs. control children (p < 0.001 uncorrected)*					
-	-	-	-	-	-

Anatomical localization, Talairach coordinates, *T *scores of significant activated voxels and cluster size of direct statistical comparisons between children with DD and control children during approximate calculation vs. approximate control condition are listed. Reported brain regions were uncorrected at p < 0.001 with a minimum number of 10 voxels.

#### ROI analysis

Although direct statistical contrasts revealed no differences in brain activation between children with or without DD in regions known to play an important role in number processing, subsequent ROI analysis indicated weaker brain activation in almost the entire network for approximate calculation in children with DD.

A total number of 14 ROIs were defined in number processing related and supporting areas. Table 1 describes all determined ROIs. The percentage of signal change (ΔS) and the mean t-values were computed within these ROIs for all subjects.

**Figure 4:**
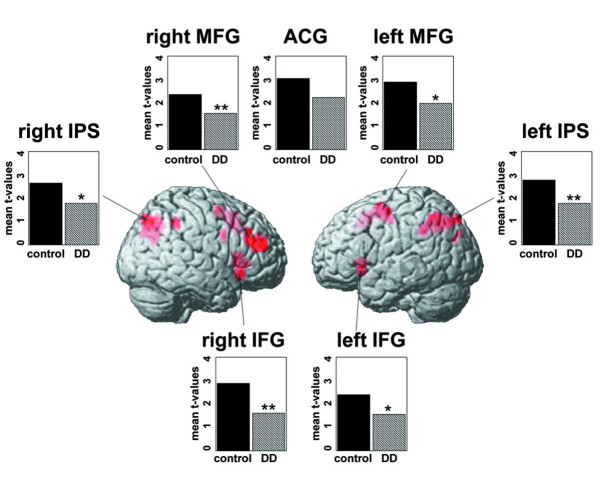
**ROI-analysis**. Mean t-values in each defined ROI for approximate calculation in control children (black) and children with DD (striped) are shown. Significant group differences are marked with two stars (** *p *< 0.05) and trends with one star (* p < 0.1).

When comparing mean ΔS and mean t-values between children with or without DD in each ROI by t-tests, significant differences or trends for differences in mean t-values were observed in six out of seven ROIs for approximate calculation (Figure 4). Children with DD exhibited significantly weaker activation in the left IPS (*p *< 0.05), right IFG (*p *< 0.05) and right MFG (*p *< 0.05) and showed a trend toward weaker activation in the right IPS (*p *< 0.1), left IFG (*p *< 0.1) and left MFG (*p *< 0.1). For exact calculation and magnitude comparison, no differences could be found in any ROI by statistical comparison of mean ΔS and mean t-values.

The IPS is the area that is always active when dealing with numbers. Accordingly, a repeated-measures GLM analysis in this region for approximate and exact calculation was conducted. Data thus obtained in the left IPS suggested the same trend observed in direct comparison between conditions and between groups. No significant differences between the conditions could be found (Wilk's Lambda F (1, 36) = 0.274, *p *< 0.7), but a trend could be observed for an interaction between condition (AP, EX) and type (children with DD, control children) (Wilk's Lambda F (1, 36) = 2.993, *p *< 0.1). This interaction suggests that differences in activation of the left IPS are greater in approximate calculation than in exact calculation when comparing children with DD with control children. Or reworded, brain activation is nearly the same during exact calculation but different during approximate calculation for the two groups. No differences between conditions and no interactions could be found in the right IPS when computing repeated-measures GLM analysis.

##### Performance contribution on ΔS and mean t-values

As mentioned above, behavioral results showed slightly lower ACC in children with DD compared to typically achieving children for approximate and exact calculation. To test the relation between performance level and brain activation, partial correlations were computed, controlled for type (with or without DD), between ACC and ΔS and mean t-values in each ROI for approximate and exact calculation. One subject was excluded from this analysis due to very low ACC levels (AP: 26%; EX: 21%). Only in approximate calculation was there a significant correlation, or a trend toward correlation. ACC in approximate calculation correlated positively with mean t-values in the right MFG (*p *< 0.05), left IPS (*p *< 0.1) and left IFG (*p *< 0.1).

## Discussion

### Differences in brain activation between children with DD and control children

The results of this study show particular differences in activation patterns between children with DD and typically achieving children. Children with DD showed greater inter-individual variance than typically achieving children and exhibited weaker activation of the arithmetic network during approximate calculation, when compared to typically achieving children.

The observed differences in brain activation could be attributed to differences in cerebral organization rather than to effects of task difficulty or quality of response, since no differences in ACC and RT between children with DD and control children were found. In addition, children of both groups reported that they could easily solve the tasks. Based on performance indicators and the individual impressions of the participants, both groups were able to perform the presented tasks. Furthermore, both groups had normal intelligence and showed no other neurological, psychiatric or learning disorders (e.g. dyslexia, ADHD), supporting the argument that the observed differences are likely to correspond to specific neuronal differences.

Akin to our findings, previous studies described differences in brain activation for calculation in adult dyscalculic patients. By activation of similar regions of dyscalculic and control subjects, abnormal modulation of brain activation was reported in dyscalculic patients [34,36]. Dyscalculic subjects with Turner's syndrome showed an insufficient recruitment of the right IPS with increasing number size [34], and dyscalculic subjects with fragile X syndrome did not show increasing activation with greater task difficulty as was observed in normal controls [36]. In addition, a single case study of a 17-year-old boy with dyscalculia and dyslexia after early brain injury associated with right parietal skull fracture and right temporal lobe hemorrhage disclosed predominantly left hemisphere activation involving frontal and parietal regions in contrast to control subjects who showed a bilateral activation pattern [37]. Studies examining impairments of the parietal-prefrontal network in fragile X syndrome subjects suggest that synaptic malfunctions may result in an impaired neuronal system that is not able to recruit a sufficient number of neurons and leads to weaker overall activation [36,55]. However, it remains still unclear, whether these differences in brain activation are specific to number processing, or whether they belie more general cognitive processing deficits in subjects with Turner's-, fragile X-syndrome or brain injury.

In addition, morphometric and spectroscopic findings corroborate a particular defect of the parietal lobe in dyscalculic subjects. A voxel-based morphometric MR study in adolescents born preterm demonstrate that children with deficits in calculation have less gray matter in the left IPS compared to those who do not have this deficit [40]. In addition, the examination of an 18-year-old man with DD using MR spectroscopy revealed a focal wedge-shaped defect in the left parieto-temporal area of the brain where a decreased signal of metabolites suggested an alteration in cell density and energetics [38]. Apart from reported left hemispheric deficits, a decrease in grey matter was found in the right parietal lobe of dyscalculic subjects with Turner's syndrome. Morphometric analysis revealed that their right hemispheric IPS was shallower and tended to be shorter [39]. Finally, findings from behavioral studies, which investigated the correlation between arithmetic dysfunction and functional brain laterality in children, also suggested that dysfunction of either hemisphere hampers arithmetic reasoning [12,56].

In view of the reported structural abnormalities in calculation-impaired subjects, the observed weaker brain activation in dyscalculic children may be a result of anatomical variability. Developmental disorders, like dyscalculia, may be accompanied by global and regional alterations in brain structure that may influence functional imaging results [57]. In general, disease or injury forces atypical structural development. Given that the BOLD response is measured within gray matter, the signal within voxels with more gray matter will be stronger. Consequently, observed differences in BOLD between children with and without DD might reflect reduced gray matter in corresponding regions. However, atypical structural development must not necessarily be observed in gray matter density. For instance, an immature or disturbed network may produce a weak change in signal because experience has not reinforced synaptic connections and thus the network is not consolidated [57]. Consolidation occurs when neuronal connections are continuously used. Prior to consolidation a neuronal network is depicted by low-level and diffuse activation.

Taken together, data from different functional, morphometric and spectroscopic MR studies as well as from behavioral studies indicate that dysfunctions, particularly in the IPS, of either hemisphere are associated with deficits in calculation. However, arithmetic ability seems to be more profoundly disturbed in subjects with left hemisphere dysfunction. This conclusion is strengthened by findings of lesion studies of the left IPS followed by specific difficulties in calculation [30,58].

Our results affirm particular differences in parietal and prefrontal brain activation between children with DD and typically achieving children. More specifically, brain activation of children with DD during approximate calculation was significantly weaker in the left IPS, right IFG and right MFG and tended to be weaker in the right IPS, left IFG and left MFG. However, no differences in brain activation could be detected during exact calculation and magnitude comparison between children with DD and control children. Furthermore, brain activation in some regions that showed weaker fMRI-signal was significantly correlated with performance measures (left IPS, left IFG, right MFG).

The fact that brain activation in children with DD was significantly weaker only in approximate calculation, but similar to controls in exact calculation and magnitude comparison, and that differences in activation between children with and without DD tended to be larger in the left IPS in approximate calculation than exact calculation, may reflect specific impairments of dyscalculic children in tasks that require an understanding of proximity relations between numbers. This is supported by the fact that exact calculation mediated by counting and fact retrieval strategies revealed no differences. In the same way, simple comparison of different quantities of objects processed by predominantly visual brain regions exposed no differences between children with DD and control children.

Findings from behavioral studies of normal development of spatial number representations and arithmetic competence contribute to the interpretation of our results. In adults, the spatially oriented mental number line is, among others, evidenced by the SNARC effect (Spatial Numerical Association of Response Codes: faster left-hand responses to small numbers and faster right-hand responses to large numbers [59]). SNARC effects arise after the 2nd grade in typically developing children and are positively correlated with arithmetic performance in boys [15,60]. Interestingly, children with visuospatial and numerical disabilities did not show SNARC effects [61]. These results indicate that visuospatial and numerical disabilities are interlinked and may be mediated by an abnormality in constructing a mental number line and representing ordinal numbers. We hypothesize that children with disturbed development of a mental number line would predominantly exhibit difficulties in arithmetic problems relying on ordinal number representations. At the same time, brain activation in regions thought to host the mental number line would be reduced in these children. Consequently, reported weaker brain activation in children with DD during approximate calculation may refer to an impaired automated access to or disordered construction of the mental number line.

A recent study provides further evidence for problems in the automatic activation of magnitudes by digits in people with DD [62]. They examined the association between Arabic numerals and the representation of magnitude by testing a Stroop-like numerical congruity effect in students with DD. Results showed that Arabic numerals do not always automatically activate their internal representation of magnitude even when attending to features that characterize magnitude (e.g. size). The authors suggest that both neuroanatomical deviations and practice might have led to a disconnection or weak connections between Arabic numerals and magnitude. Furthermore, Koontz and Berch [63] found that children with DD have problems in subitizing (determining the magnitude of a small set of items), which is considered to be an automated process. Taken together, all of these behavioral studies highlight a specific impairment of an automated access to analog magnitude representation of numbers in dyscalculic subjects.

Given that children with DD showed weaker activation only during approximate calculation, but not during the other two tasks let us suggest that observed differences are not simply a matter of anatomical variability. If a dissociation is observed such that group differences occur for one cognitive function but not for others, then it can be presumed that true functional differences exist [57].

Finally, it has to be pointed out that a direct contrast of brain activation patterns in children with DD and controls revealed no differences in any condition when correcting for multiple comparisons. Only when using uncorrected thresholds was stronger activation in the right insula and the right parahippocampal gyrus observed in control children compared to children with DD during approximate calculation. Both regions are not expected to be specifically task-related. Only comparison of mean t-values in ROIs that were defined by activated clusters in the control group revealed differences between children with DD and control children. It has to be considered that if a region is selected on the basis that it is shown to be activated in one group (here, the control group), then comparison with any other group statistically inflates the chances that increased activation in the first group will be found. However, in the present study both groups showed positive activation in all ROIs and activation patterns of typically achieving children should mirror the ordinary cerebral representation of number processing enabling age-appropriate arithmetic efficiency.

### Brain activation patterns in children with DD and control children

In general, children with DD showed similar brain activation patterns during approximate calculation, exact calculation and magnitude comparison when compared to typically achieving children. This is in accordance with fMRI studies that investigated brain activation in dyscalculic subjects suffering from Turner's-, fragile X-syndrome or early brain injury. All of them reported similar activated regions both in subjects with DD and in unaffected subjects [34,36,37]. The activation sites for children with DD and typically achieving children fall within brain areas that are well known to be involved in arithmetic processing in non-impaired adults and children, including parietal and prefrontal areas [19-24,64,65].

When contrasting approximate and exact calculation, our findings did not result in a functional dissociation of these two calculation tasks neither in children with DD nor in control children, also demonstrated by Dehaene et al. for adults [20]. Only when using uncorrected thresholds were differences between approximate and exact addition found for typically achieving children. For instance, control children activated more strongly the anterior cingulate gyrus during approximate calculation compared to exact addition, which reflects more the higher attention and working memory load during approximation rather than a dissociation between the quantity based and verbal system for number processing. A recent study also reported no significant differences between approximate and exact calculation using either non-symbolic or symbolic stimuli [33]. Venkatraman et al. hypothesized that it is difficult for participants to perform mental arithmetic approximately, which is particularly true for simple arithmetic where individuals automatically compute the exact result. Furthermore, Burbaud and colleagues [66] could show that brain activation of the quantity based and verbal system varied between subjects due to individual strategy choice rather than condition. Taken together, our results may indicate a particular difficulty in study design to clearly disentangle these two distinct processing routes, but they do not reject the existence of a dissociated route for quantity based and verbal number processing. Approximate and exact addition conditions are probably too similar to evoke clear differences in brain activation by direct comparison in a group of subjects. Furthermore, inter-individual differences in strategy choice also have an important impact on brain activation patterns.

## Conclusion

In summary, the present study presents the first attempt at characterizing the neural underpinnings of DD in otherwise typically achieving schoolchildren. Results indicate differences between children with DD and controls in approximate calculation with dyscalculic children exhibiting weaker activation in almost the entire neural network. In particular, the left IPS, left IFG and right MFG seem to play a crucial role in correct number processing, since brain activation correlated with accuracy rate in these regions. Nevertheless, dyscalculic and typically achieving children in general activated similar neural networks during number processing.

To conclude, these group differences appear to reflect a deficiency in recruitment of neural resources designated for the processing of analog number magnitudes in dyscalculic children. In contrast, no differences in brain activation could be detected for exact calculation mediated by arithmetic fact retrieval and for non-symbolic magnitude comparison. These results may indicate a difficulty in establishing an abstract spatial number representation as predicted from neuropsychological research (SNARC-effect).

Future research is needed to answer remaining questions concerning school instruction and therapeutic issues, i.e. which kind of special training may optimize and induce specific brain plasticity in children with DD. Furthermore, the ambitious but promising step from neuroscience into the classroom has to be taken [67].

*Abbreviations*: ACC = accuracy rate, ACG = anterior cingulate gyrus, AP = approximate calculation, DD = developmental dyscalculia, EX = exact calculation, FDR = false discovery rate, FG = fusiform gyrus, fMRI = functional magnetic resonance imaging, FWE = family wise error, GLM = general linear model, IFG = inferior frontal gyrus, IOG = inferior occipital gyrus, IPL = inferior parietal lobe, IPS = intraparietal sulcus, ITG = inferior temporal gyrus, LG = lingual gyrus, MC = magnitude comparison, MFG = middle frontal gyrus, MOG = middle occipital gyrus, rCBF = regional cerebral blood flow, RT = reaction time, SFG = superior frontal gyrus, SPL = superior parietal lobe, ΔS = signal change

## Competing interests

The author(s) declare that they have no competing interests.

## Authors' contributions

KK has been involved in developing the study design, has programmed the paradigm, recruited subjects, carried out all fMRI examinations and data analysis, had a major role in the interpretation of data and prepared the present manuscript.

TL has been involved in developing the study design, gave substantial technical support in data acquisition and analysis, reviewed the manuscript critically for important intellectual content and gave final approval of the version to be published.

TD has been involved in developing the design of the paradigm to be tested and gave support in data analysis and interpretation.

MD performed the evaluation of neuropsychological reports and was involved in the coordination of appointments for fMRI-testing.

EM has been involved in developing the study design, reviewed the manuscript critically for important intellectual content and gave final approval of the version to be published.

MvA has been involved in developing the study design, made substantial contributions to concepts of data interpretation, reviewed the manuscript critically for important intellectual content and gave final approval of the version to be published.

## References

[B1] Butterworth B (2005). The development of arithmetical abilities. J Child Psychol Psychiatry.

[B2] Kaufmann L, Lochy A, Drexler A, Semenza C (2004). Deficient arithmetic fact retrieval-storage or access problem? A case study. Neuropsychologia.

[B3] Temple CM (1991). Procedural dyscalculia and number fact dyscalculia: Double dissociation in developmental dyscalculia. Cogn Neuropsychol.

[B4] Landerl K, Bevan A, Butterworth B (2004). Developmental dyscalculia and basic numerical capacities: a study of 8-9-year-old students. Cognition.

[B5] WHO (2005). ICD-10. International Statistical Classification of Diseases and Related Health Problems 10th Revision; Chapter V: Mental and behavioral disorders (F81.2).

[B6] Shalev RS, Auerbach J, Manor O, Gross-Tsur V (2000). Developmental dyscalculia: prevalence and prognosis. Eur Child Adolesc Psychiatry.

[B7] Lewis C, Hitch GJ, Walker P (1994). The prevalence of specific arithmetic difficulties and specific reading difficulties in 9- to 10-year-old boys and girls. J Child Psychol Psychiatry.

[B8] Shalev RS, Manor O, Gross-Tsur V (2005). Developmental dyscalculia: a prospective six-year follow-up. Dev Med Child Neurol.

[B9] Ardila A, Rosselli M (2002). Acalculia and dyscalculia. Neuropsychol Rev.

[B10] Monuteaux MC, Faraone SV, Herzig K, Navsaria N, Biederman J (2005). ADHD and dyscalculia: Evidence for independent familial transmission. J Learn Disabil.

[B11] Reiss AL, Eliez S, Schmitt JE, Patwardhan A, Haberecht M (2000). Brain imaging in neurogenetic conditions: realizing the potential of behavioral neurogenetics research. Ment Retard Dev Disabil Res Rev.

[B12] Rosenberg PB (1989). Perceptual-motor and attentional correlates of developmental dyscalculia. Ann Neurol.

[B13] Shalev RS (2004). Developmental dyscalculia. J Child Neurol.

[B14] Alarcon M, DeFries JC, Light JG, Pennington BF (1997). A twin study of mathematics disability. J Learn Disabil.

[B15] Schweiter M, Weinhold Zulauf M, von Aster MG (2005). Die Entwicklung räumlicher Zahlenrepräsentationen und Rechenfertigkeiten bei Kindern. Zeitschrift für Neuropsychologie.

[B16] Dellatolas G, von Aster MG, Willadino-Braga L, Meier M, Deloche G (2000). Number processing and mental calculation in school children aged 7 to 10 years: a transcultural comparison. Eur child Adolesc Psychiatry.

[B17] Shalev RS, Gross-Tsur V (2001). Developmental dyscalculia. Pediatr Neurol.

[B18] Shalev RS, Manor O, Kerem B, Ayali M, Badichi N, Friedlander Y, Gross-Tsur V (2001). Developmental dyscalculia is a familial learning disability. J Learn Disabil.

[B19] Burbaud P, Degreze P, Lafon P, Franconi JM, Bouligand B, Bioulac B, Caille JM, Allard M (1995). Lateralization of prefrontal activation during internal mental calculation: a functional magnetic resonance imaging study. J Neurophysiol.

[B20] Dehaene S, Spelke E, Pinel P, Stanescu R, Tsivkin S (1999). Sources of mathematical thinking: behavioral and brain-imaging evidence. Science.

[B21] Menon V, Rivera SM, White CD, Glover GH, Reiss AL (2000). Dissociating prefrontal and parietal cortex activation during arithmetic processing. NeuroImage.

[B22] Pesenti M, Thioux M, Seron X, De Volder A (2000). Neuroanatomical substrates of arabic number processing, numerical comparison, and simple addition: a PET study. J Cogn Neurosci.

[B23] Rickard TC, Romero SG, Basso G, Wharton C, Flitman S, Grafman J (2000). The calculating brain: an fMRI study. Neuropsychologia.

[B24] Schmithorst VJ, Brown RD (2004). Empirical validation of the triple-code model of numerical processing for complex math operations using functional MRI and group Independent Component Analysis of the mental addition and subtraction of fractions. NeuroImage.

[B25] Dehaene S, Piazza M, Pinel P, Cohen JD (2003). Three Parietal Circuits for Number Processing. Cogn Neuropsychol.

[B26] Dehaene S (1992). Varieties of numerical abilities. Cognition.

[B27] Dehaene S, Cohen JD (1995). Towards an anatomical and functional model of number processing. Mathematical Cognition.

[B28] Stanescu-Cosson R, Pinel P, van De Moortele PF, Le Bihan D, Cohen L, Dehaene S (2000). Understanding dissociations in dyscalculia: a brain imaging study of the impact of number size on the cerebral networks for exact and approximate calculation. Brain.

[B29] Lemer C, Dehaene S, Spelke E, Cohen L (2003). Approximate quantities and exact number words: dissociable systems. Neuropsychologia.

[B30] Takayama Y, Sugishita M, Akiguchi I, Kimura J (1994). Isolated acalculia due to left parietal lesion. Arch Neurol.

[B31] Dehaene S, Cohen L (1997). Cerebral pathways for calculation: double dissociation between rote verbal and quantitative knowledge of arithmetic. Cortex.

[B32] Cohen L, Dehaene S, Chochon F, Lehéricy S, Naccache L (2000). Language and calculation within the parietal lobe: a combined cognitive, anatomical and fMRI study. Neuropsychologia.

[B33] Venkatraman V, Ansari D, Chee MW (2005). Neural correlates of symbolic and non-symbolic arithmetic. Neuropsychologia.

[B34] Molko N, Cachia A, Riviere D, Mangin JF, Bruandet M, Le Bihan D, Cohen L, Dehaene S (2003). Functional and structural alterations of the intraparietal sulcus in a developmental dyscalculia of genetic origin. Neuron.

[B35] van Harskamp NJ, Rudge P, Cipolotti L (2002). Are multiplication facts implemented by the left supramarginal and angular gyri?. Neuropsychologia.

[B36] Rivera SM, Menon V, White CD, Glaser B, Reiss AL (2002). Functional brain activation during arithmetic processing in females with fragile X Syndrome is related to FMR1 protein expression. Hum Brain Mapp.

[B37] Levin HS, Scheller J, Rickard T, Grafman J, Martinkowski K, Winslow M, Mirvis S (1996). Dyscalculia and dyslexia after right hemisphere injury in infancy. Arch Neurol.

[B38] Levy LM, Reis IL, Grafman J (1999). Metabolic abnormalities detected by 1H-MRS in dyscalculia and dysgraphia. Neurology.

[B39] Molko N, Cachia A, Riviere D, Mangin JF, Bruandet M, LeBihan D, Cohen L, Dehaene S (2004). Brain anatomy in Turner syndrome: evidence for impaired social and spatial-numerical networks. Cereb Cortex.

[B40] Isaacs EB, Edmonds CJ, Lucas A, Gadian DG (2001). Calculation difficulties in children of very low birthweight: a neural correlate. Brain.

[B41] Kucian K, Loenneker T, Dietrich T, Martin E, von Aster MG (2005). Development of Neural Networks for Number Processing: an fMRI Study in Children and Adults [abstract]. NeuroImage.

[B42] WMA (2002). The World Medical Association’s Declaration of Helsinki: Ethical Principles for Medical Research Involving Human Subjects.

[B43] von Aster MG (2001). ZAREKI (Neuropsychological Test Battery for Number Processing and Calculation in Children).

[B44] Kaufman AS, Kaufman NL, al. DFPM (1994). Kaufman-assessment battery for children (K-ABC).

[B45] Wechsler D, al. UT (1999). Hamburg-Wechsler-Intelligenztest für Kinder III (HAWIK-III) / Übersetzung und Adaption der WISC-III von David Wechsler.

[B46] Klassencockpit (2004). MS_Deutsch_61, MS_Mathematik_61.

[B47] Marx H (1998). Knuspels Leseaufgaben. Gruppenlesetest für Kinder Ende des ersten bis vierten Schuljahres..

[B48] Landerl K, Wimmer H, Moser E (1997). Salzburger Lese- und Rechtschreibtest. Verfahren zur Differentialdiagnose von Störungen des Lesens und Schreibens für die 1. bis 4. Schulstufe.

[B49] (2002). SPSS for Windows, Statistical Product and Service Solution.

[B50] Genovese CR, Lazar NA, Nichols T (2002). Thresholding of statistical maps in functional neuroimaging using the false discovery rate. NeuroImage.

[B51] Lancaster JL, Woldorff MG, Parsons LM, Liotti M, Freitas CS, Rainey L, Kochunov PV, Nickerson D, Mikiten SA, Fox PT (2000). Automated Talairach atlas labels for functional brain mapping. Hum Brain Mapp.

[B52] Talairach J, Tournoux P (1988). Co-planar Stereotaxic Atlas of the Human Brain: 3-Dimensional Proportional System: An Approach to Cerebral Imaging.

[B53] Cohen J (1988). Statistical Power Analysis for the Behavioral Sciences.

[B54] D'Amico EJ, Neilands TB, Zambarano R (2001). Power analysis for multivariate and repeated measures designs: a flexible approach using the SPSS MANOVA procedure. Behav Res Methods Instrum Comput.

[B55] Kwon H, Menon V, Eliez S, Warsofsky IS, White CD, Dyer-Friedman J, Taylor AK, Glover GH, Reiss AL (2001). Functional neuroanatomy of visuospatial working memory in fragile X syndrome: relation to behavioral and molecular measures. Am J Psychiatry.

[B56] Shalev RS, Manor O, Amir N, Wertman-Elad R, Gross-Tsur V (1995). Developmental dyscalculia and brain laterality. Cortex.

[B57] Berl MM, Vaidya CJ, Gaillard WD (2006). Functional imaging of developmental and adaptive changes in neurocognition. NeuroImage.

[B58] Martins IP, Ferreira J, Borges L (1999). Acquired procedural dyscalculia associated to a left parietal lesion in a child. Child Neuropsychol.

[B59] Dehaene S, Bossini S, Giraux P (1993). The mental representation of parity and number magnitude. J Exp Psychol.

[B60] Berch DB, Foley EJ, Hill RJ, McDonough Ryan P (1999). Extracting Parity and Magnitude from Arabic Numerals: Developmental Changes in Number Processing and Mental Representation. J Exp Child Psychol.

[B61] Bachot J, Gevers W, Fias W, Roeyers H (2005). Number sense in children with visuospatial disabilities: orientation of the mental number line. Psychology Science.

[B62] Rubinsten O, Henik A (2005). Automatic activation of internal magnitudes: a study of developmental dyscalculia. Neuropsychology.

[B63] Koontz KL, Berch DB (1996). Identifying Simple Numerical Stimuli: Processing Inefficiencies Exhibited by Arithmetic Learning Disabled Children. Mathematical Cognition.

[B64] Kawashima R, Taira M, Okita K, Inoue K, Tajima N, Yoshida H, Sasaki T, Sugiura M, Watanabe J, Fukuda H (2004). A functional MRI study of simple arithmetic--a comparison between children and adults. Brain Res Cogn Brain Res.

[B65] Rivera SM, Reiss AL, Eckert MA, Menon V (2005). Developmental Changes in Mental Arithmetic: Evidence for Increased Functional Specialization in the Left Inferior Parietal Cortex. Cereb Cortex.

[B66] Burbaud P, Camus O, Guehl D, Bioulac B, Caille J, Allard M (2000). Influence of cognitive strategies on the pattern of cortical activation during mental subtraction. A functional imaging study in human subjects. Neurosci Lett.

[B67] Gura T (2005). Educational research: big plans for little brains. Nature.

